# Differentiating Parkinson Disease From Traumatic Encephalopathy Syndrome

**DOI:** 10.1212/NE9.0000000000200092

**Published:** 2023-09-25

**Authors:** Nita Chen, Lauren Fanty, Ariane Veilleux Carpentier, Michael S. Okun

**Affiliations:** From the Department of Neurology, University of Florida, Gainesville.

The recent publication detailing Muhammad Ali's clinical diagnosis of levodopa-responsive young-onset Parkinson disease^1^ has provided an educational opportunity for clinicians and trainees to enhance their diagnostic acumen, especially when encountering cases with a history of head trauma. A clinical diagnosis of Parkinson disease is usually made in patients with progressive bilateral asymmetric levodopa-responsive features accompanied by motor and nonmotor symptoms with or without resting tremor, with or without a history of traumatic brain injury. By contrast, progressive cognitive impairment after multiple repetitive impacts to the head, with or without neurobehavioral dysregulation, with or without a postural action tremor (which may be transitory), more commonly leads to a diagnosis of traumatic encephalopathy syndrome. This teaching aid can be used to highlight these important features and to illustrate that it is possible for the syndromes to co-occur inclusive of their respective pathologies. Finally, all clinicians should appreciate that head trauma is a risk factor for the later occurrence of Parkinson disease (Figure).

**Figure:**
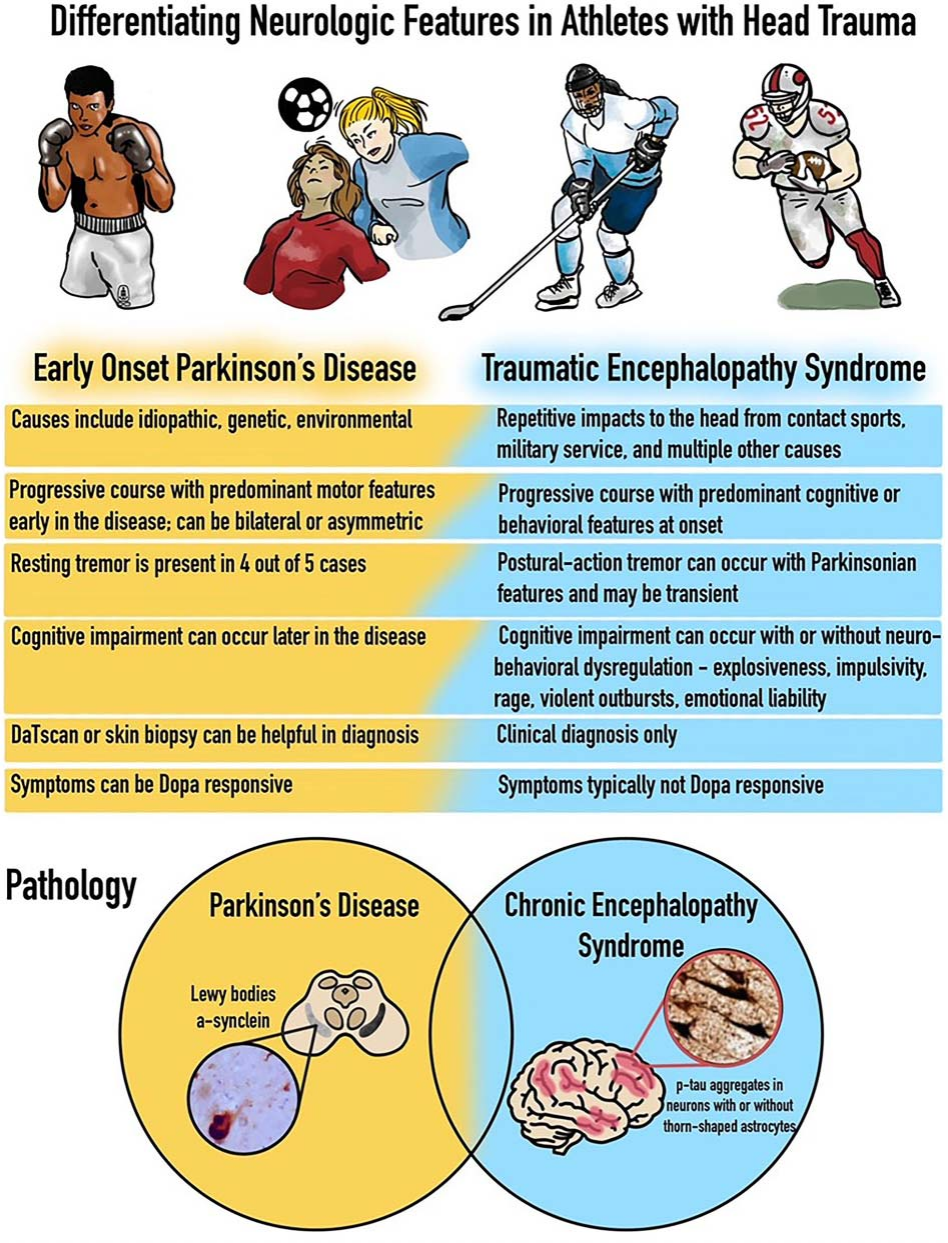
Features That Can Be Used by Clinicians to Try to Differentiate Early-Onset Parkinson Disease From Traumatic Encephalopathy Syndrome in Athletes or in Persons With a History of Head Trauma To move from a diagnosis of CTE syndrome to CTE will require pathology. CTE pathology is commonly characterized by hyperphosphorylated tau (p‐tau) protein, which is present as neurofibrillary tangles, astrocytic tangles, and neurites, and these tend to cluster around smaller blood vessels in the cortex and commonly are observed at sulcal depths. CTE pathology is common in the frontal and medial temporal lobes, as well as the thalamus and brainstem. TDP-43 pathology also commonly occurs in most cases with CTE. CTE = chronic traumatic encephalopathy; TDP-43 = TAR DNA–binding protein–43.

## Appendix Authors

**Table:**

Name	Location	Contribution
Nita Chen, MD	Department of Neurology, University of Florida, Gainesville	Drafting/revision of the article for content; including medical writing for content; major role in the acquisition of data
Lauren Fanty, MD, MPH	Department of Neurology, University of Florida, Gainesville	Drafting/revision of the article for content; including medical writing for content
Ariane Veilleux Carpentier	Department of Neurology, University of Florida, Gainesville	Drafting/revision of the article for content; including medical writing for content
Michael S. Okun, MD	Department of Neurology, University of Florida, Gainesville	Drafting/revision of the article for content; including medical writing for content

## References

[1] Okun MS, Mayberg HS, DeLong MR. Muhammad Ali and young-onset idiopathic Parkinson disease: the missing evidence. JAMA Neurol. 2023;80(1):5-6. doi:10.1001/jamaneurol.2022.3584 36279117

